# Structure and Property Evolution of Microinjection Molded PLA/PCL/Bioactive Glass Composite

**DOI:** 10.3390/polym17070991

**Published:** 2025-04-06

**Authors:** Meiqiong Chen, Yinghong Chen, Haihao He, Xinwen Zhou, Ning Chen

**Affiliations:** National Key Laboratory of Advanced Polymer Materials, Polymer Research Institute of Sichuan University, Chengdu 610065, China; xca2023@163.com (M.C.); hehaihao@stu.scu.edu.cn (H.H.); 2023223090071@stu.scu.edn.cn (X.Z.); ningchen@scu.edu.cn (N.C.)

**Keywords:** microinjection molding, polylactic acid, polycaprolactone, bioactive glass, biocomposite, in situ fiber formation

## Abstract

In this study, the microinjection molding technology was adopted to prepare polylactic acid (PLA)/polycaprolactone (PCL)/bioactive glass (BG) composites with varying BG contents for biomedical applications. The various measurement techniques, including scanning electronic microscopy (SEM), X-ray diffraction (XRD), Fourier transform infrared (FT-IR) spectroscopy, the water contact angle (WCA) test, the mechanical test, and in vitro biological evaluations, were applied to characterize the above interesting biocomposites. The experimental results show that the extremely strong shear force field generated during the microinjection molding process could induce the in situ formation of micron PCL dispersed phase fibril structures and strongly promote the homogeneous dispersion of micron BG filler particles in the PLA/PCL polymer matrix, which therefore leads to a significant improvement in the specific mechanical property of the PLA/PCL/BG composite. For example, with BG fillers content increasing to 10 wt%, the Young’s modulus of the above obtained PLA/PCL/BG composite could reach 2122.9 MPa, which is 1.47 times higher than that of the unfilled PLA/PCL blend material. In addition, it is also found that under the simulated body fluid (SBF) environment, the incorporated BG fillers in the PLA/PCL polymer matrix could be effectively transformed into hydroxyapatite (HA) components on the treated sample surface, thus being greatly advantageous to enhancing the material’s in vitro bioactivity. Obviously, the microinjection molded PLA/PCL/BG biocomposites could exhibit excellent comprehensive performance, revealing that the microinjection molding processing method could hold great potential in industrialization applications of the resulting biodegradable biomedical materials.

## 1. Introduction

The metallic materials such as stainless steel [1], cobalt–chromium alloys [2], and titanium-based alloys [3] are extensively utilized in orthopedic implants due to their favorable processability and superior mechanical characteristics. These metallic materials have demonstrated effective structural support in early orthopedic surgeries, particularly in those regions which could bear high mechanical loads, such as the hip joint, knee joint, and spine. However, these materials suffer from some limitations, such as biological inertness (in bone tissue integration and regeneration) [4], the stress shielding effect [5], metal ion release, and cytotoxicity [6]. Obviously, conventional metal implants cannot degrade in vivo, and a secondary surgery is required to remove the implants after bone healing [7]. This could not only exacerbate the patient pain but also significantly elevate the risk of postoperative complications such as infections and secondary fractures.

In recent years, with increasing demand for bone repair materials, bioresorbable polymer materials have emerged as promising alternatives to traditional metal implants [8,9]. The ideal bone repair materials must possess mechanical properties that are comparable to those of natural bone, as well as osteoinductive properties [10]. However, bioresorbable polymers are not sufficiently bioactive and exhibit poor cell adhesion and affinity, which consequently hinders the effectiveness of bone regeneration [11]. Moreover, single bioresorbable polymers also could not provide adequate osteoinductivity or osteoconductivity, thus further limiting their applications in bone defect repair. To overcome these challenges, the design and development of bioresorbable polymer/bioceramic composites have become the critical focus of current studies. Despite the excellent bioactivity of bioceramics, their brittleness and poor processability greatly restrict their applications in some load-bearing sites, and the strategy for construction of composite materials can significantly improve the deficiencies of single bioceramic materials [12]. Particularly, by being composited with bioresorbable polymers, the ceramic particles dispersed in the flexible polymer matrix could effectively enhance the material’s mechanical strength while still preserving the favorable processability of the polymer. Obviously, combining the ductility and processability of polymers with the bioactivity of ceramic fillers can well achieve the complementary and optimized properties of the prepared polymer biocomposites.

Among the various bioceramic fillers, bioactive glass (BG) is regarded as one of the class A bioactive materials with unique advantages [13]. Compared with other bioceramic fillers that only possess osteoconductivity, such as hydroxyapatite (HA) [14,15], β-tricalcium phosphate (β-TCP) [16], and calcium phosphate (CaP) [17], BG could exhibit additional osteoinductivity [18]. The osteoinductive properties of BG mainly originates from its solubilization properties in vivo. When BG is in contact with body fluids, it can release ionic products such as Si^2+^, Ca^2+^, and P^5+^, which can directly interact with cells, and promote the differentiation of undifferentiated mesenchymal stem cells (MSCs) into osteoblasts by modulating the expression of osteogenesis-related genes, thus accelerating bone tissue regeneration and repair. Except for the promotion in bone regeneration via ion release, BG has another key advantage due to its rapid ability to deposit bone-like mineralized components. When a sample containing BG is exposed to body fluids, a layer of hydroxyapatite (HA), which has a similar composition to natural bone tissue, would be formed on the sample’s surface within hours to days, significantly reducing the osteogenic bonding time between the material and the host tissue [19]. With its excellent osteoinductive properties, BG could offer unique advantages in bone regeneration, making it highly applicable across a wide range of applications. On the other hand, for the potential polymer matrices to be considered for the preparation of the filled biocomposites, polylactic acid (PLA) and polycaprolactone (PCL) are two kinds of famous bioabsorbable polymers which receive extensive attention and are broadly applied due to their low cost, ease of processing, and excellent biocompatibility. Among these two polymers, PLA is featured due to its high rigidity and brittleness, whereas PCL is noted for its flexibility. So, both polymers have strong complementary properties, and this makes them suitable for the fabrication of PLA/PCL-based filled composites with balanced mechanical properties. Several relevant experiments were performed, and the resulting composites could be applied in medical areas [20,21,22,23].

Up to now, there have been several strategies applied to realize the processing and forming of BG filler-filled polymer biocomposites, such as 3D printing [24,25], electrostatic spinning [26,27], compression molding [28], conventional injection molding [29], and so on. However, the above-mentioned processing methods can generally be used to fabricate some composite parts with a bigger size and a macro-scale structure, and it is difficult to manufacture some small-size parts (such as micro bone screws, micro vascular clamps, and microneedles) with a micro-scale structure (particularly in the range of micrometer). On the other hand, in the above-mentioned processing methods, the involved shear rate of melt during processing is very low. As far as the regular injection molding process is concerned, its shear rate is generally at the 10^3^ S^−1^ level, and the shear rate of the other processing methods is lower than that of the regular injection molding method. Under such low shear rate conditions, it is generally difficult to realize the formation of highly oriented structures in the polymer matrix, such as the fibrillar structure and the shish-kebab structure, thus leading to limited performance enhancement. Comparatively, microinjection molding technology is a special advanced processing method, which is completely different from the regular injection molding technology due to the great reduction in the size of the runner and microcavity. The microinjection molding process proves to possess an extremely high shear rate (as high as 10^6^ S^−1^, i.e., a very strong shear stress field) [30], which is advantageous for the in situ formation of the highly oriented reinforced structures [9,31,32], as it promotes more homogeneous filler dispersion [33] and enhances the interfacial interaction and compatibility between the fillers and the polymer matrix [34], thus resulting in the high performance of the composite’s microparts. In addition, microinjection molding could also provide a much higher injection pressure, injection speed, temperature gradient, forming precision, batch production capacity, and shorter filling time [35]. It can achieve the batch and automatic production of biomedical microdevices with high dimensional accuracy [36]. The detailed comparisons between different processing methods are presented in Supporting Information (Table S1). So, microinjection molding could be an ideal processing strategy to fabricate the high-performance biomedical microparts of bioresorbable polymers/BG composites. However, unfortunately, similar studies have never been performed so far. It is interesting to conduct the microinjection molding of bioresorbable polymers/BG composites and investigate the corresponding structure and property change, which could lay a good foundation for developing scalable micro bone repair implants for biomedical applications.

Accordingly, in this study, the PLA/PCL/BG ternary composite system was first constructed, where the PLA/PCL blend was used as the polymer matrix (mainly considering that both polymers have complementary mechanical performances, good processability, and most importantly, relatively low costs, as mentioned before), BG was used as the bioactive ceramic filler dispersed phases, and PCL was applied as the polymer dispersed phases to toughen the total composite system. Then, the microinjection molding of the prepared PLA/PCL/BG ternary composite was carried out. Taking advantage of the extremely strong shear stress field generated during microinjection molding, the in situ fibrillation of PCL dispersed phases and the homogeneous dispersion of micron BG ceramic filler particles in the polymer matrix could be expectedly achieved. The effects of microinjection molding processing on the structure and properties of the prepared PLA/PCL/BG biocomposites were explored. On this basis, the morphology characterization, mechanical performance, hydrophilic property, and biological property of the microinjection-molded PLA/PCL/BG composites were carefully performed and investigated. The related investigations could help to provide fundamental data and novel biomedical polymer composites for the scalable fabrication of high-performance bone repair microdevice implants with good bioactivity (bone regeneration ability), high efficiency, and low costs.

## 2. Materials and Methods

### 2.1. Material

The raw material Nature Works 4032D polylactic acid (PLA) with a density of 1.24 g/cm^3^ and a melting temperature of about 170 °C was bought from Nature Works (Blair, NE, USA). Perstorp Capa6800 polycaprolactone (PCL) with a number-averaged molecular weight (*M_n_*) of 80,000 g/mol was supplied by Perstorp (Malmö, Sweden). The micron bioactive glass filler particles (RTS-KY58) were obtained from Foshan JinKeLan Biotechnology Co., (Guangdong, Foshan, China). The simulated body fluid (SBF, 1.5×, sterile, CZ0403) was provided by the Beijing Leagene Biotech. Co., Ltd. (Beijing, China).

### 2.2. Sample Preparation

**Figure 1:**
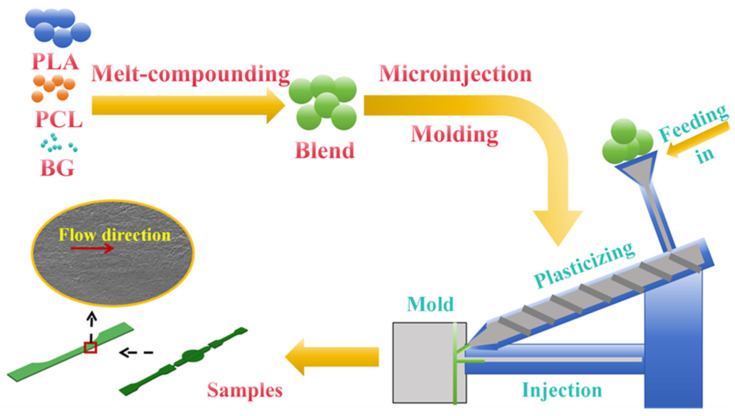
The schematic diagram for the preparation of the PLA/PCL blend and PLA/PCL/BG composite microparts.

Pure PLA and pure PCL were initially dried in a vacuum oven at 80 °C and 40 °C for 8 h, respectively. After the drying process, the mixture of PLA, PCL, and BG fillers was homogeneously mixed in a high-speed mixer, where the weight ratio of PLA to PCL was set to 70:30, and the weight fraction of BG fillers was set to 0 wt%, 5 wt%, 10 wt%, and 15 wt%, respectively. The well-mixed blend of PLA, PCL, and BG fillers was then melt-compounded in a torque internal mixer (model RM-200C, Harbin Harp Electric Technology Co., Ltd., Harbin, China) under a condition of a temperature of 180 °C, a rotor speed of 35 rpm, and a mixing time of 5 min. The cooled melt-compounded PLA/PCL/BG composite cakes were then pulverized into small pieces, which were further dried in a vacuum oven at 45 °C for 8 h to eliminate residual moisture. These dried small pieces were subsequently added to a Wittmann–Battenfeld MicroPower 5 microinjection molding machine (GmbH, Kottingbrunn, Austria) and injection-molded into micro tensile parts for various tests. The specific microinjection molding processing conditions used include a melt temperature of 180 °C, an injection speed of 400 mm/s, an injection pressure of 1500 bar, and a cooling time of 6 s. The preparation process is illustrated in Figure 1.

### 2.3. Sample Characterization

An Apreo S HiVac field emission scanning electronic microscope (SEM, FEI, Hillsboro, OR, USA) was employed to examine the morphology of the BG filler particles and the fractured surface of the microinjection-molded micro tensile sample at a 5 kV accelerating voltage. Before observation, the microinjection-molded micro tensile sample was cryo-fractured in liquid nitrogen while being parallel to and also perpendicular to the melt flow direction, respectively. Then, a thin gold layer was deposited on the fractured surfaces of the micro tensile samples and the surface of the BG filler particles via vacuum spraying, respectively. Meanwhile, the attached Octane Elect Super energy dispersive spectroscopy (EDS) machine was used to perform the local elemental analysis of the sample surface. The particle size and its distribution of BG fillers were determined using an S3500 laser diffraction particle size and image analyzer (Microtrac MRB, Montgomeryville, PA, USA) with an ultrasonic frequency of 2000 Hz. Before the measurement, the BG filler particles were ultrasonically dispersed in ethanol for 5 min. A model AR2000ex flat plate rheometer (TA, New Castle, DE, USA) was used to test the dynamic rheological properties of the samples. The rheological test samples were hot press-molded disks with a diameter of 25 mm and a thickness of 1 mm. The test conditions were as follows: a temperature of 180 °C, a strain amplitude of 1%, and a scanning frequency of 0.1–100 Hz. X-ray diffraction (XRD) was applied to investigate the crystalline structure of the samples at room temperature. The analyses were conducted on an Ultima IV X-ray diffractometer (Rigaku, Tokyo, Japan) with a voltage of 40 kV and a current of 40 mA using Cu-Kα radiation. The diffraction range was 2θ = 10–80°, and the scanning speed was 10°/min. The Fourier transform infrared (FTIR) spectrometer (Nicolet iS50, Thermo Fisher Scientific, Waltham, MA, USA) was used to identify the functional groups of the involving samples (including the BG filler sample and the PLA/PCL/BG composite sample). For the former, the BG filler particles and KBr were evenly mixed using a mortar and then compressed into a transparent disk sample for the test. The testing conditions were set over a scanning range of 400–4000 cm^−1^, a resolution of 4 cm^−1^, and a number of scans of 32 times. The tensile properties of the PLA/PCL/BG composite were measured on a micro tensile sample by using a 5567 universal material testing machine (Instron Corporation, Norwood, MA, USA) at a cross-head speed of 2 mm/min, and the averaged value of 5 parallel specimens was used. The water contact angle was measured using a DSA30 optical contact angle tester (KRÜSS GmbH, Hamburg, Germany) at room temperature, with a measurement range of 0–180° and a resolution of 0.01°. Each sample was measured five times, and the average value was calculated. Finally, the in vitro bioactivity of the microinjection-molded PLA/PCL/BG composite was measured. Monitoring hydroxyapatite (HA) formation in the simulated body fluid (SBF) for different samples could be used to indirectly assess their bioactivities [37]. Firstly, the microinjection-molded samples were cut into 0.5 cm × 0.5 cm square slices to ensure that their dimensions satisfy the requirements for the subsequent immersion and analysis. The samples were fully submerged in a 1.5× concentrated SBF solution with a ratio of SBF volume to biomaterial surface area of 10 mL/cm^2^ [28]. The SBF solution was kept at 37 °C, with the immersion time set at 3, 7, 14, and 21 days, respectively. To ensure experimental accuracy, the SBF solution was refreshed every 72 h. After each predetermined immersion time was reached, the involved sample was removed, rinsed with deionized water, and then dried in a vacuum oven at 60 °C for 24 h before subsequent characterization. The resulting HA formation on the sample surface was further evaluated using SEM, EDS, XRD, and FTIR analyses, respectively.

## 3. Results and Discussion

### 3.1. Characterization of BG Particles

**Figure 2:**
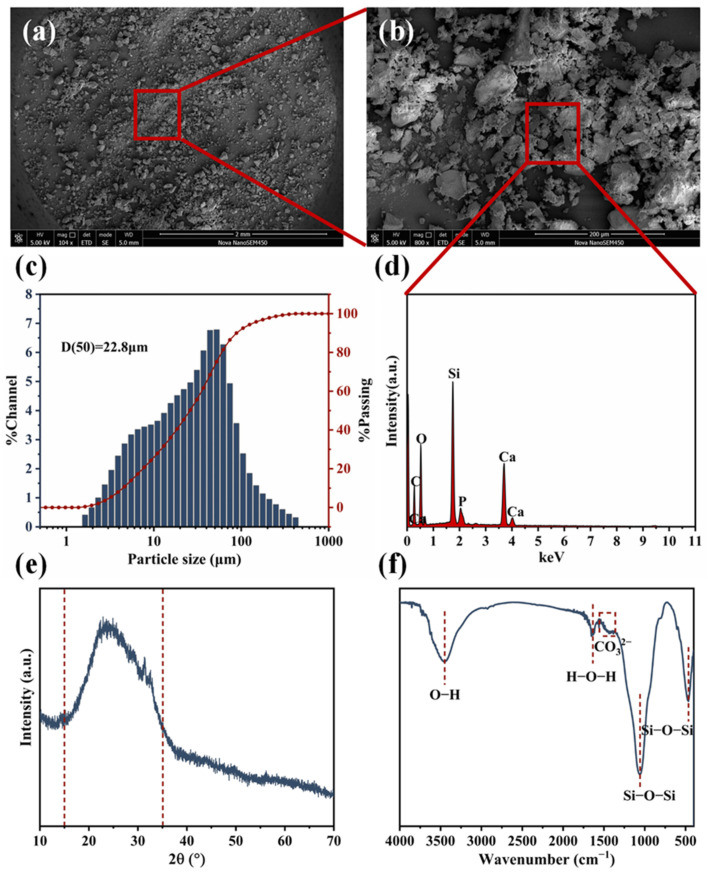
The SEM micrographs (**a**,**b**), particle size distribution result (**c**), EDS elemental analysis result (**d**), XRD pattern (**e**), and FT-IR spectrum (**f**) of pure BG filler particles.

The morphological characteristics and elemental composition of the BG filler particles are shown in Figure 2. The SEM morphology results indicate that the incorporated BG fillers have a micron particle size and an irregular particle shape (Figure 2a), and they also have a rough surface (Figure 2b) and an uneven particle size distribution (Figure 2c). The EDS elemental analysis results show that the main elements of C, O, Si, P, and Ca exist in the BG particles (Figure 2d). The laser particle size analysis result reveals that there is a particle size distribution of the BG filler particles in the range of 5–180 μm, with an average size of 22.8 μm (Figure 2c). The XRD measurement was also performed on the BG fillers. The obtained XRD patterns show that there is a broad peak occurring between 2θ = 15° and 35° (Figure 2e), which is the characteristic of amorphous SiO_2_, indicating an amorphous structure in the incorporated BG fillers. In the FTIR spectra of BG particles, there are absorption peaks occurring at 1059 cm^−1^ and 466 cm^−1^ (Figure 2f), which are corresponding to the stretching and bending vibrations of the Si-O-Si functional group, respectively. Additionally, the absorption peaks at 3450 cm^−1^ and 1653 cm^−1^ represent the stretching vibrations of the -OH group and bending vibrations of the H-O-H group of BG fillers after water absorption [38]. The weak absorption peaks occurring in the range of 1500–1300 cm^−1^ are attributed to the possible formation of CO_3_^2−^ groups after the CO_2_ absorption performed by BG fillers [26,39].

### 3.2. Rheological Properties

**Figure 3:**
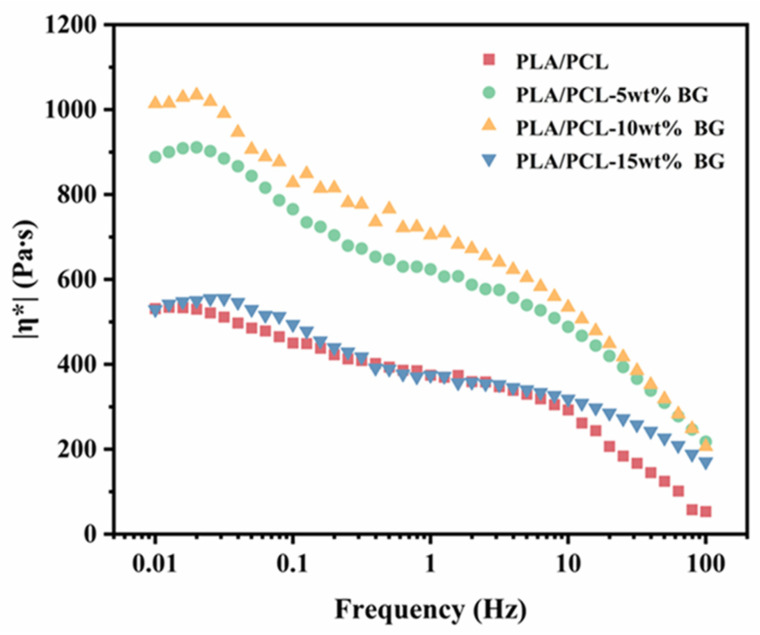
The frequency dependency of complex viscosity (η*) for the PLA/PCL blend and PLA/PCL/BG composites with different amounts of BG content.

During thermal processing, the melt flow behavior of polymers is critical to the forming quality of the final product. Especially for microinjection molding, this involves a small runner and cavity size, as well as an extremely rapid cooling rate. Figure 3 shows the rheological response results of the PLA/PCL blend and PLA/PCL/BG composites with varying amounts of BG content. As can be seen, for the PLA/PCL blend, the viscosity remains relatively constant in the low-frequency region as the shear rate increases, but at a higher shear rate, the viscosity decreases rapidly. This behavior could be attributed to insufficient shear stress at the low shear rate, which cannot cause the disentanglements of macromolecular chains, thus resulting in an almost stable viscosity in the low-frequency region. However, at the higher shear rate, the macromolecular chains become disentangled, thus leading to the rapid decrease in viscosity, particularly in the high-frequency region (in the range of >10 Hz), i.e., showing the shear thinning behavior. For the BG filler-incorporated composite system, it is found that there is a slight increase in viscosity observed in the low-frequency region. This increase could be attributed to the physical cross-linking effect of BG particles (the hydrogen bonding interactions between the hydroxyl groups on the surface of BG fillers and the ones on PLA polymers), which possibly prevents the movements of PLA and PCL macromolecular chains to a certain degree. As the BG content increases (particularly rising to 10 wt%), the viscosity of the composites exhibits a significant increasing tendency. Such the change could be attributed to the enhanced physical hindrance of BG particles on the slippage and flow of polymer molecular chains, as well as the increased hydrogen bonding interactions between BG particles and PLA polymer matrix, further restricting matrix polymer melt flowability [40]. However, when the BG content further increases to 15 wt%, the viscosity of the composite system sharply decreases to a level comparable to that of the PLA/PCL blend system. Such a sudden change is very strange. It is believed that the reason for this could be very likely linked to the catalytic degradation effect of the excessive part of BG fillers added (>10 wt% loading) on PLA under high-shear and high-temperature processing conditions (the primary reason), as revealed in the subsequent TG measurement results. The incorporated BG filler particles at a big loading may lower the activation energy of PLA’s thermal degradation reaction, thereby accelerating the degradation process and significantly reducing PLA’s molecular weight [41,42]. The thermal degradation behaviors of the prepared PLA/PCL/BG composites with various amounts of BG content were investigated by using TG measurements (Supporting Information with Figure S1). Additionally, the structural heterogeneity and non-homogenous mixing of fillers (agglomeration) at higher BG loading (e.g., 15 wt%) may be a secondary consideration for the decrease. As a result, the above factors both contribute to the obvious decrease in the material’s viscosity.

### 3.3. Morphology Analysis of Dispersed Phases in the Polymer Matrix

**Figure 4:**
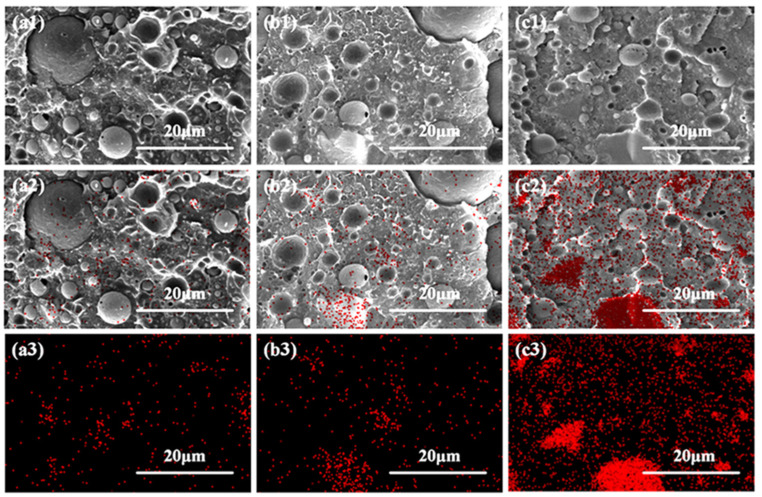
The SEM micrographs (**a1**,**a2**,**b1**,**b2**,**c1**,**c2**) and EDS analysis results (distribution of red points shown in **a2**,**a3**,**b2**,**b3**,**c2**,**c3**) of the cryo-fractured surface of simply melt-compounded PLA/PCL/BG composites (along being perpendicular to the melt flow direction) with 5 wt% BG fillers (**a1**–**a3**), 10 wt% BG fillers (**b1**–**b3**), and 15 wt% BG fillers (**c1**–**c3**), respectively. (**a2**,**b2**,**c2**) are the combination of SEM images shown in (**a1**,**b1**,**c1**), with EDS results shown in (**a3**,**b3**,**c3**), respectively. The location of red points represents the distribution of Si (EDS results), reflecting the dispersion of BG fillers.

**Figure 5:**
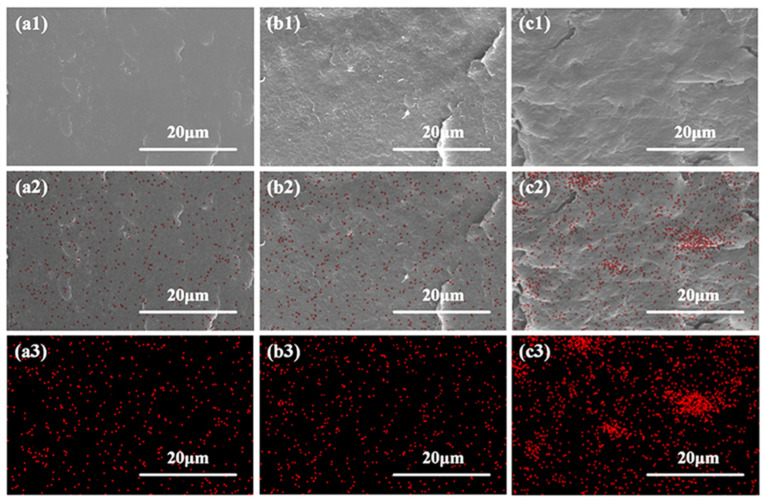
The SEM micrographs (**a1**,**a2**,**b1**,**b2**,**c1**,**c2**) and EDS analysis results (**a2**,**a3**,**b2**,**b3**,**c2**,**c3**) of the cryo-fractured surface of microinjection-molded PLA/PCL/BG composites (along being perpendicular to the melt flow direction) with 5 wt% BG fillers (**a1**–**a3**), 10 wt% BG fillers (**b1**–**b3**), and 15 wt% BG fillers (**c1**–**c3**), respectively. (**a2**,**b2**,**c2**) are the combination of SEM images shown in (**a1**,**b1**,**c1**) with EDS results shown in (**a3**,**b3**,**c3**)**,** respectively. The location of red points represents the distribution of Si (EDS results), reflecting the dispersion of BG fillers.

Generally, the properties of one composite are largely dependent on the dispersion of the fillers within the matrix [43,44] and also the internal phase morphology structure [34]. In order to explore whether the microinjection molding process could facilitate the in situ formation of PCL fibrillar structures and also the uniform dispersion of BG particles within the polymer matrix, SEM, as well as EDS characterization, was employed to analyze the internal morphology and elemental distribution of the PLA/PCL blend and PLA/PCL/BG composite with varying amounts of BG content prepared through simply melt compounding in an internal mixer (Figure 4) and microinjection molding (Figure 5), respectively. As shown in Figure 4 (SEM images), the melt-compounded composites exhibit a typical and clear “sea-island” structure. There are many spherical particles of all sizes (as “islands”) distributed in the polymer matrix (as “sea”). According to the irregular shape of the BG filler particles (Figure 2a,b) and the EDS analysis results (much less red points on the spherical particles shown in Figure 4a2,b2,c2), it could be believed that the above spherical particles are formed from the PCL dispersed phases. From Figure 4, it can be further found that the size of these PCL spherical particles is very different (from 2 μm to 18 μm), and interfacial gaps between the PCL spherical particles and the matrix are also clearly observed. The above results show that the PCL spherical particles are poorly dispersed in the polymer matrix, and the interfacial compatibility is also poor. For the dispersed phase of the BG filler, as seen from the SEM photos of Figure 4 at varying amounts of BG content, the size of the BG filler particles is remarkably reduced under the effect of the shear force of the internal mixer (compared with the SEM images of BG particles shown in Figure 2), and the interfacial compatibility between the BG filler particles and the PLA matrix remains good due to the presence of hydrogen bonding interactions. However, due to presence of two dispersed phases (PCL and BG), it is not easy to distinguish the distribution of BG inorganic filler particles in the matrix. Accordingly, EDS was adopted to evaluate the content of silicon (Si, the primary element of BG) in various microdomains of the sample, which can be used to assess the distribution of BG filler particles within the material. The EDS results indicate that the dispersion of BG filler particles is not good in the polymer matrix due to the very weak shear force field exerted by the internal mixer and exhibits a tendency to agglomerate. As the BG content increases, the degree of agglomeration becomes remarkably increased, suggesting the increasingly deteriorated dispersion of the BG filler particles within the matrix (particularly for the 15 wt% BG-filled sample).

In order to investigate the influence of microinjection molding processing on the morphology evolution of dispersed phases in the PLA/PCL/BG composite, the morphology and elemental distribution of the microinjection-molded PLA/PCL/BG composite that is perpendicular to the melt flow direction were investigated, and the results are shown in Figure 5. As can be seen, the morphology of the microinjection-molded sample is very different from that of the simply melt-compounded sample. Compared with the latter, the PCL spherical particles of all sizes disappear in the former. Instead, in the fractured surface of the microinjection-molded samples with varying BG contents, many small spheres of PCL dispersed phases with a remarkable size reduction are formed and evenly dispersed in the matrix resin. These PCL dispersed phases exhibited a tendency to form a co-continuous two-phase structure with the PLA matrix. The substantial decrease in the dimension of PCL dispersed phases could be caused by the extremely strong shear stress field generated during microinjection molding processing. For the dispersed phases of BG filler particles, the EDS analysis results (Figure 5a2,a3,b2,b3,c2,c3), which could indirectly well reflect their distribution, reveal that the dispersion of BG filler particles within the matrix is significantly improved. Especially for the samples with BG contents of 5 wt% and 10 wt%, the BG filler particles are more uniformly distributed. When the BG content increases to 15 wt%, although there is agglomeration of a small amount of BG particles still occurring, the extent of the agglomeration becomes notably decreased in total compared to the simply melt-compounded sample. The significant improvement in the dispersion of BG fillers is also ascribed to the extremely strong shear stress field of microinjection molding. As a result, the microinjection molding technology could effectively enhance the dispersion of BG fillers in the PLA/PCL polymer matrix, thereby optimizing the microstructure of the composite materials.

**Figure 6:**
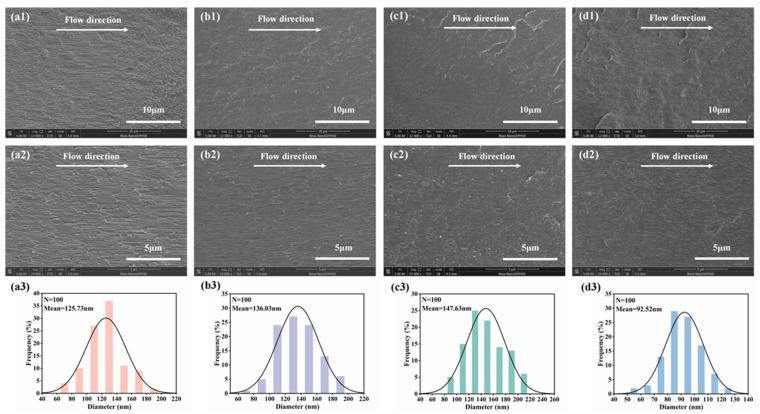
The SEM images of the cryo-fractured surfaces and the radial size distribution of the in situ-formed fibers of the PLA/PCL blend micropart (**a1**–**a3**) and the PLA/PCL/BG composite micropart with 5 wt% BG fillers (**b1**–**b3**), 10 wt% BG fillers (**c1**–**c3**), and 15 wt% BG fillers (**d1**–**d3**), respectively, along the melt flow direction.

Figure 6 shows the SEM morphology photos and the particle size distribution of dispersed phases of the microinjection-molded PLA/PCL blend sample and microinjection-molded PLA/PCL/BG composite samples with different amounts of BG content along the melt flow direction. As can be seen, in the microinjection-molded PLA/PCL blend sample, the resulting strong shear force could induce the in situ formation of clear fibrillar structures within the matrix [45], which are oriented along the flow direction. The radial size distribution of these fibers formed could be obtained using the Nano Measure 1.2.5 software. After measurement and calculation, the radial size of the in situ-formed fibers in the PLA/PCL blend could achieve an average value of approximately 125.7 nm. When the BG content increases to 5 and 10 wt%, the formed fibrillar structures show a layered distribution, with BG filler particles uniformly interspersed in the matrix and encapsulated by the fibers, thus enhancing the interfacial compatibility between BG and the PLA/PCL polymer matrix [34]. In addition, it is also seen that the particle size of the BG particles is significantly smaller than their original average particle size, indicating that the extremely strong shear forces exerted by the microinjection molding would make the BG particles fragment into smaller sizes, which can further facilitate their dispersion in the matrix. The slight increase in the fiber radial size and the decrease in the fiber length with the higher BG content are likely due to the elevated viscosity of the higher loading of the BG fillers [46,47], which restricts the stretching of melt during fiber formation under the effect of the strong shear stress field. However, at 15 wt% BG loading, there is an agglomeration of a small amount of BG particles occurring in some regions (higher BG loading would result in more difficult filler dispersion), which negatively influences fiber encapsulation, thus weakening the interfacial compatibility. It is also found that the average fiber diameter decreases significantly, from 147.6 nm to 92.5 nm. The reason for this may be due to accelerated PLA degradation in the presence of higher loading of BG fillers under high-temperature processing conditions. The incorporated BG fillers with a higher content could possibly reduce the activation energy of PLA’s thermal degradation to a greater degree [41,42], thus leading to a decrease in molecular weight and much lower viscosity, and hence the reduced fiber diameter size.

### 3.4. Mechanical Property Analysis

**Figure 7:**
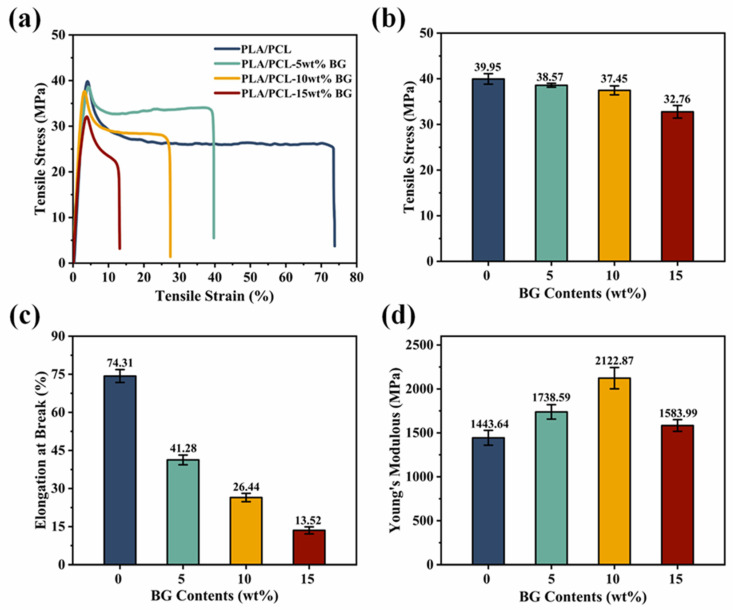
The stress–strain curve (**a**), tensile strength (**b**), elongation at the break (**c**), and Young’s modulus (**d**) of the PLA/PCL blend micropart and PLA/PCL/BG composite microparts with different BG contents.

The mechanical properties of the microinjection-molded part of the PLA/PCL blend and the PLA/PCL/BG composite with different amounts of BG content were evaluated by measuring the tensile stress–strain curves, with results indicated in Figure 7. As can be seen, all the different samples exhibit well-defined stress–strain curves with a clear yielding phenomenon, which could be explained by good BG filler dispersion and in situ PCL fibrillation within the PLA/PCL matrix. As the rigid inorganic filler, BG can significantly enhance the filled composite microparts’ Young’s modulus, e.g., when BG loading is increased from 0 wt% to 10 wt%, the corresponding Young’s modulus would also increase from 1443.6 MPa to 2122.9 MPa. However, with the BG loading further increasing to 15 wt%, the corresponding Young’s modulus would decline to 1566.2 MPa, which is primarily attributed to the incorporation of higher amounts of BG fillers, thereby inducing PLA degradation during high-temperature microinjection molding processing [42,48], which compromises mechanical performance (a decrease in molecular weight would certainly decrease the material’s stiffness). In addition, the agglomeration of the BG particles at a higher loading may be another reason for impairing their Young’s modulus (stiffness). Furthermore, by increasing the BG content, the tensile strength of the composite microparts presents a slightly decreasing tendency (in the range of 0–10 wt% BG loading), followed by a significantly decreasing tendency (in the range of higher than 10 wt% BG loading). The first slow decrease result at a lower BG content (5 wt% and 10 wt% BG loading) is likely due to the uniform distribution of BG particles, good interfacial compatibility, and the formation of PCL fibrillar structures. In contrast, by increasing the BG content to 15 wt%, the resulting tensile strength decreases significantly to 32.8 MPa. The reason for the decline could be ascribed to the agglomeration of BG filler particles, impaired PCL fibrillation at higher BG concentrations, and the effects of polymer degradation. The involving BG agglomeration would lead to the stress concentration within the material, thereby limiting the effective stress transfer from the matrix to the fillers [49] and hence leading to an increase in the sample’s brittleness and a reduction in the sample’s tensile strength. It is also noted that the elongation at the break continuously decreases as the BG filler content increases, and this is primarily due to the restricting effect of the incorporated rigid BG fillers on the polymer macromolecular chains’ movement, as well as polymer degradation, thus diminishing the sample’s tensile deformability. Overall, while moderate BG loading (5 wt% and 10 wt%) could significantly improve the Young’s modulus and tensile strength of the material, the excessive BG content (15 wt%) would lead to filler agglomeration, stress concentration, and impaired PCL fibrillation, thus ultimately deteriorating the material’s overall mechanical properties. Considering that the incorporation of BG fillers would negatively influences the mechanical properties to different extents, the microinjection-molded PLA/PCL/BG biocomposites are expected to be applied in the bone repair of non-load-bearing or low load-bearing parts of the body (e.g., cartilage repair).

### 3.5. Hydrophilic Property Analysis

**Figure 8:**
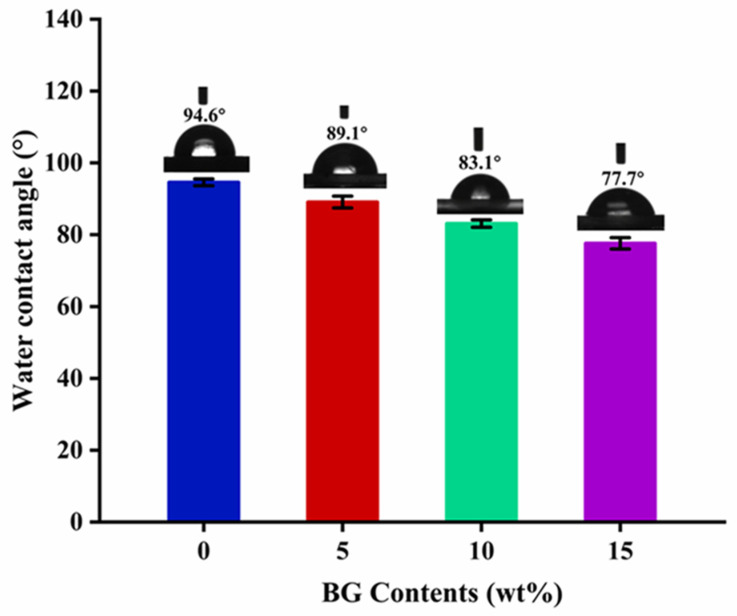
The water contact angles of the neat PLA/PCL blend and PLA/PCL/BG composites with different BG contents.

Hydrophilicity plays a crucial role in enhancing cell adhesion on material surfaces, with higher hydrophilicity generally promoting cell attachment [50]. The water contact angle measurements of the PLA/PCL blend and PLA/PCL/BG composites with different BG contents are shown in Figure 8. As can be seen, the water contact angle of the unfilled PLA/PCL blend exceeds 90°, indicating that this blend material is highly hydrophobic [47]. So, hydrophobicity is typically unfavorable for cell adhesion and proliferation, as a larger contact angle suggests a reduced capability of the material surface to combine with water, thus negatively influencing the formation of a liquid film necessary for cell attachment. By increasing the BG content, the water contact angle of the samples shows a constantly decreasing tendency, and it reaches 77.7° at 15 wt% BG loading. The decrease in water contact angle can be mainly attributed to the BG’s inherent hydrophilicity derived from its abundance of surface hydroxyl groups, which contribute a significant number of hydrophilic active sites, thus resulting in the improved material’s hydrophilicity [19]. Furthermore, as the BG content rises, the surface roughness of the material also increases, which could provide the necessary sites for water droplet adsorption. These roughened surfaces could aid in the spreading of water molecules, thereby further helping reduce the water contact angle and enhance the material’s overall hydrophilicity [39]. On the other hand, another possible less significant reason may be related to the degradation of polymer chains at higher BG loading (because the decrease in polymer molecular weight would also cause the reduction in hydrophobicity [51]). Compared to the other PCL/BG composites reported in the literature (with water contact angles typically ranging from 80° to 90°) [52,53,54], the composite with 15 wt% BG content in this study could exhibit superior hydrophilicity, as evidenced by its lower water contact angle of 77.7°.

### 3.6. In Vitro Bioactivity Analysis

**Figure 9:**
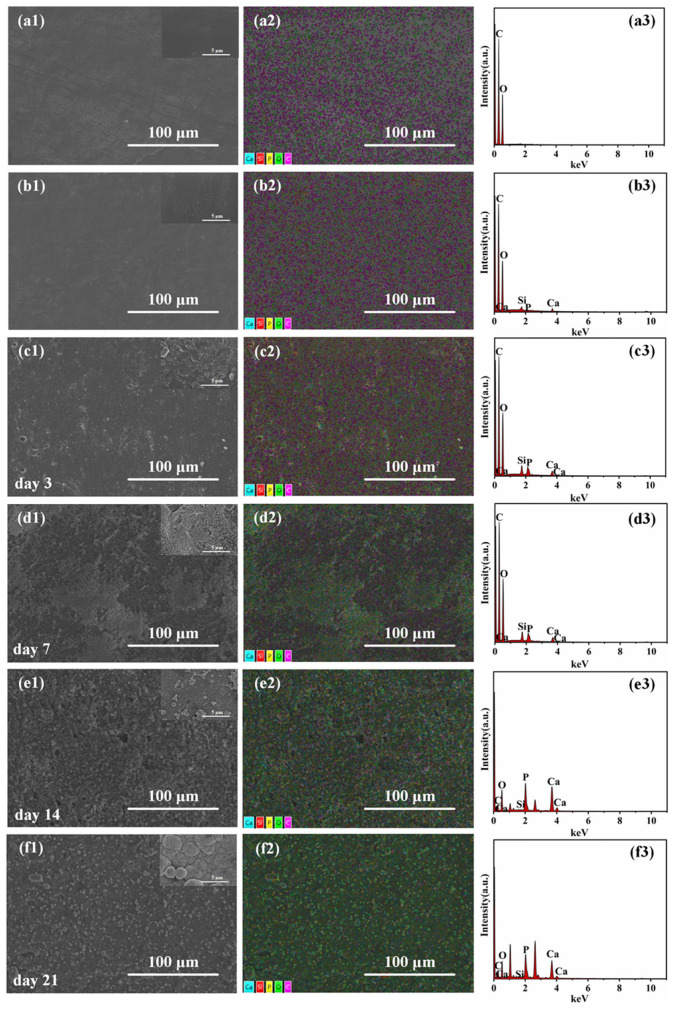
The sample surface’s SEM and EDS results of the PLA/PCL blend (**a1**–**a3**), the untreated PLA/PCL/10 wt% BG composite (**b1**–**b3**), and the treated PLA/PCL/10 wt% BG composite in SBF for 3 days (**c1**–**c3**), 7 days (**d1**–**d3**), 14 days (**e1**–**e3**), and 21 days (**f1**–**f3**), respectively.

The bioactivity of the microinjection-molded PLA/PCL/10 wt% BG composite micropart was assessed by immersing it in the SBF solution at 37 °C for different amounts of time (3, 7, 14, and 21 days) to examine the formation of hydroxyapatite (HA) on the composite micropart surface. The surface morphology changes and elemental composition were analyzed using SEM as well as the attached EDS characterizations, and the results are illustrated in Figure 9. It can be seen that the surface of PLA/PCL/BG composites is rougher than that of the PLA/PCL blend and exhibits the existence of Si, Ca, and P elements distribution, which is absent in the PLA/PCL blend. It is believed that when the PLA/PCL/10 wt% BG composite micropart sample interacts with the simulated body fluid (SBF), HA formation would occur on the surface through ionic reactions. As HA is a primary component of bones, it can improve the cell adhesion and proliferation property [55]. After 3 days of immersion, the HA substances were initially found to be formed on the sample surface, although the formation rate is limited. The above result demonstrates that the ions released from the interaction of BG fillers with SBF could initiate HA deposition. With an extension of the immersion time (to 7–21 days), the deposited HA particles significantly increase. At 7 days, the formed HA layers become thickened and progressively cover the total sample surface. At 14 days, the surface of the material was almost completely covered by the formed HA layers, and typical cauliflower-like HA crystal particles start to appear, which is the structural feature commonly found during the HA mineralization process. At 21 days, the HA crystals grow to be impinged and would finally form the dense HA layers that could help to improve the bioactivity of BG fillers, thus creating a more favorable surface for cell attachment and proliferation. In addition, from the SEM images, it is also found that there are cavities on the sample surface as the immersion time increases. These cavities are likely caused by BG degradation [29] and hence help to increase the contact area with SBF, thereby promoting HA mineralization. The presented EDS analysis results show a marked rise in Ca and P levels on the sample surface with the extension of the immersion time, while the Si content reveals a gradually decreasing trend. The above results indicates that the incorporated BG fillers are gradually consumed and converted into HA deposits. By 14 days, the determined Ca/P ratio of the sample surface deposits ranges from 1.6 to 1.7, which is much close to the natural bone apatite ratio (1.67), and this further verifies the formation of HA layers in the system [56]. In the future, we will perform further bioactivity evaluations of in vitro cell experiments and in vivo implant assays on the prepared PLA/PCL/BG biocomposite microparts for better biomedical applications.

**Figure 10:**
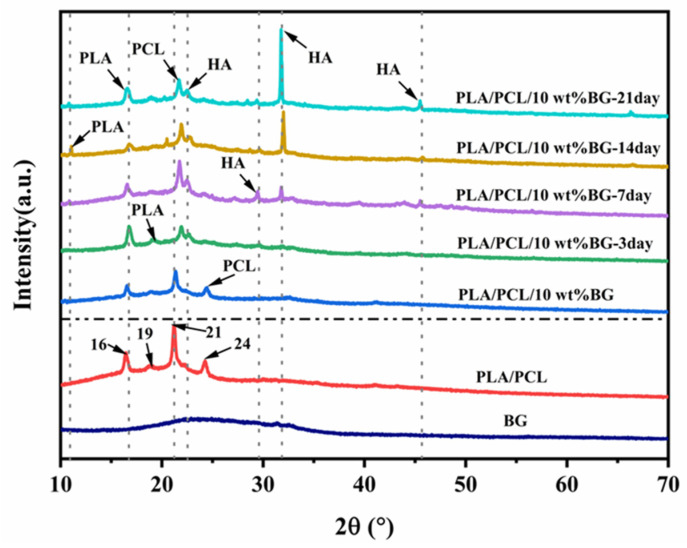
The XRD patterns of the PLA/PCL blend and the PLA/PCL/10 wt% BG composite treated in SBF for different amounts of time.

**Figure 11:**
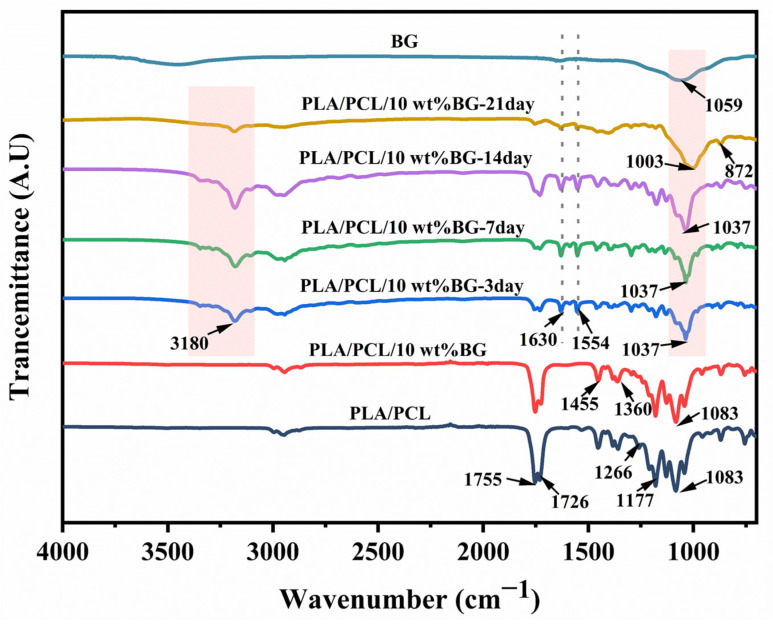
The FT-IR spectra of the PLA/PCL blend micropart and the PLA/PCL/10 wt% BG composite micropart treated in SBF for different amounts of time.

The formation of HA substances on the PLA/PCL/10 wt% BG composite sample surface was further confirmed through XRD and FTIR analyses, and the results are shown in Figure 10 and Figure 11, respectively. From the literature [57,58], it is known that the featured diffraction peaks of HA can be found at 2θ = 23° (111), 29° (210), 32° (211), and 46° (222). As can be seen from Figure 10, in the XRD patterns of the PLA/PCL blend and the untreated PLA/PCL/BG composite, two diffraction peaks occur at 2θ = 16° and 19°, corresponding to the α crystalline conformation of PLA [59], as well as a pair of peaks at 2θ = 21° and 24°, corresponding to the PCL crystal structure [60]. After immersion in SBF for 3 days, three new weak peaks appear at 2θ = 23°, 29°, and 32°, respectively, which are the diffraction peaks from HA [61,62], indicating the initial formation of HA substances [61]. By increasing the immersion time to 7 days, except for the reflection peaks of HA detected at 2θ = 23° (111), 29° (210), and 32° (211), there is another diffraction peak appearing at 2θ = 46°, which corresponds to the (222) reflection of HA [29]. The above results show that after 7 days of immersion treatment, HA substances could be completely formed. With the immersion time continuously prolonging (e.g., to 14 and 21 days), the intensity of the HA characteristic peaks also exhibits a significantly increasing tendency (particularly at 21 days), reflecting the mineralization evolution of the HA layers on the sample surface. In addition, there was a new diffraction peak at 2θ = 10.5° since the 7 days of immersion treatment, indicating the change in the PLA conformation, which may result from the rearrangement of molecular chains upon PLA degradation, thereby forming a new PLA crystalline structure [63,64].

Figure 11 shows the FTIR spectra of the PLA/PCL blend micropart and the PLA/PCL/10 wt% BG composite micropart treated with SBF for different amounts of time. As can be seen, there are minimal differences between before and after BG incorporation, indicating that the addition of BG fillers does not change the fundamental structure characteristics of the PLA/PCL-based material. The primary vibrational absorption peaks are consistent with the published literature [65,66], including the C=O stretching vibrations of PLA and PCL at 1755 cm^−1^ and 1726 cm^−1^, respectively; the bending vibrations of the -CH_3_ and -CH- groups at 1455 cm^−1^ and 1360 cm^−1^; the C-O-C- and C-O-related vibrations at 1177 cm^−1^ and 1130 cm^−1^; the stretching vibration of the -C(=O)-O- ester group at 1260 cm^−1^; and the symmetric stretching vibration peak of the C-O group at 1083 cm^−1^. After the immersion treatment in SBF, there are some new additional peaks emerging in the FTIR spectra: The broad absorption band at 3180 cm^−1^ and the absorption peak at 1630 cm^−1^ are corresponding to the stretching vibration of the O-H group and the bending vibration of the H-O-H group, respectively [39,67]. The peaks at 1545 cm^−1^ and 873 cm^−1^ are attributed to the asymmetric stretching vibration and out-of-plane bending vibration of CO_3_^2−^, respectively [68,69]; as the immersion time increases, the stretching vibration peak of the Si-O-Si group around 1037 cm^−1^ gradually broadens. This phenomenon may be attributed to the overlap between the Si-O-Si vibration peak and the asymmetric stretching vibration peak of PO_4_^3−^, which typically appears at 1022 cm^−1^ [69]. The above XRD and FTIR analysis results could jointly further confirm that the mineralized deposits on the sample surface are HA substances, thus proving that the incorporated BG fillers could help to enhance the in vitro bioactivity of PLA/PCL-based composite microparts to a considerable degree.

## 4. Conclusions

The PLA/PCL/BG composite microparts with varying BG contents were fabricated using microinjection molding technology, and the effects of the microinjection molding processing method on the structure and properties of the composites were systematically investigated. The morphology characterizations reveal that the microinjection molding method could facilitate the in situ formation of the PCL dispersed phase fibrillar structures, making the composite microparts transformed from the “sea-island” structures to the distinct fibrous structures. The elemental analyses further show that the microinjection molding method could effectively promote the uniform dispersion of BG filler particles within the polymer matrix by taking advantage of its extremely strong shear stress field. The mechanical property test results indicate that while the incorporation of BG filler particles could reduce the sample’s toughness, it can significantly enhance the sample’s Young’s modulus. However, due to the local agglomeration of BG filler particles at higher loading, the sample’s Young’s modulus could be negatively impacted. The in vitro bioactivity tests demonstrate that the incorporation of BG fillers could significantly improve the bioactivity of the composite micropart, as the HA layers, which are made of a similar component as the natural bone tissue, would be formed on the sample surface when the sample is exposed to the simulated body fluid (SBF) environment. Based on the above results, the microinjection molding strategy could show a great potential for the low-cost and efficient production of biomedical microdevices with biodegradability and bone regeneration potential.

## Data Availability

Data are contained within the article and Supplementary Materials.

## Supplementary Materials

The following supporting information can be downloaded at: https://www.mdpi.com/article/10.3390/polym17070991/s1, Table S1: The comparisons between different processing methods; Figure S1: The TG (a) and DTG (b) curves of pure PLA/PCL blend and PLA/PCL/BG composites with various BG content (PCL content was fixed at 30 wt%). Refs. [70,71,72,73,74,75] are cited in Supplementary Materials file.
