# Supplementary material for: Structure and Property Evolution of Microinjection Molded PLA/PCL/Bioactive Glass Composite
Source: Polymers (Basel). 2025 Apr 6;17(7):991. doi: 10.3390/polym17070991 (PMC11991442; doi:10.3390/polym17070991)
Supplement: Supplementary file 1 [file polymers-17-00991-s001.zip › polymers-3542560-supplementary.pdf]

Supporting Information

Structure and Property Evolution of Microinjection Molded PLA/PCL/Bioactive Glass Composite

Meiqiong Chen, Yinghong Chen *, Haihao He, Xinwen Zhou and Ning Chen

National Key Laboratory of Advanced Polymer Materials, Polymer Research Institute of Sichuan University, Chengdu 610065, China

* Corresponding author: Yinghong Chen (e-mail address: johnchen@scu.edu.cn)

Experimental part

TG measurement: the samples were taken from the narrow middle section of the micro-tensile microparts and analyzed using a TG209F1 thermogravimetric analyzer (NETZSCH, Selb, Germany) under N_2 atmosphere to measure the mass change as a function of temperature. Approximately 7 mg of sample was tested over a temperature range of 45–600 °C with a heating rate of 10 °C/min.

Results and Discussion

Table S1 The comparisons between different processing methods

Processing method	Shear rate level	Dimension of molded part	Forming precision	Bulk production efficiency
Microinjection molding	10^3~10^6 s^{-1}	Small/Micron size	High	High
Conventional injection molding	≤10^4 s^{-1}	Large/Medium/Small size	Medium	High
Compression molding	Near zero	Large/Medium/Small size	Low	Medium
FDM 3D printing	≤10^3 s^{-1}	Large/Medium/Small size	Medium	Medium
Electrostatic spinning	≤10^4 s^{-1}	Medium/Small size	Medium	Low

Thermogravimetric (TG) analysis

TG measurement was performed to investigate the degradation behavior of the BG fillers incorporated PLA/PCL samples, and the results are shown in Fig. S1. As can be seen, the pure PLA/PCL blend sample exhibits a single-stage thermal decomposition weight loss process. However, upon addition of BG fillers, the thermal decomposition behavior of the material undergoes a significant change, i.e. displaying a multi-stage decomposition pattern. In addition, as the BG content increases, the initial degradation temperature of the composite also gradually decreases, indicating that the incorporation of BG fillers promotes the initial polymer matrix degradation. It is interestingly found that with the degradation progressing for different content of BG fillers filled sample, the thermal stability of higher content of BG sample would gradually surpass that of lower content of BG sample. The higher the BG content, the earlier the transformation would occur (at lower temperature). In addition, the resulting DTG curves reveals that the pure PLA/PCL blend sample primarily exhibits two degradation peaks, which are corresponding to the thermal decomposition of PLA (366 °C) [70] and PCL (405 °C) [71], respectively. However, in the first decomposition stage, with incorporation of BG fillers, the resulting PLA degradation peak shifts toward a lower temperature. With further increasing BG content, the corresponding peak temperature and peak intensity would become lower and stronger, respectively, i.e. the maximum PLA degradation rate increases at higher BG content. This means that addition of BG fillers could really accelerate the earlier PLA degradation. It should be noted that in the resulting DTG curves of different samples, the degradation peaks experience the different changes at different BG content. For pure PLA/PCL blend sample, there are only two peaks occurring at 366 °C and 405°C, which are attributed to PLA and PCL degradation, respectively. However, with incorporating BG fillers, there are 3-4 degradation peaks occurring, where two of them are new ones, which are located at 350 °C and 400 °C, respectively. Particularly, the peak at 350 °C would significantly weaken and even disappear with the BG content increasing to 10-15 wt%. Obviously, the peak at 400 °C is caused by the decomposition of BG fillers. The appearance of peak at 350 °C is strange because it could not be attributed to the degradation of any individual component of the PLA/PCL/BG composite. After careful analysis, it is believed that the occurrence of peak at 350 °C could be likely related to the reaction of PLA/PCL polymer matrix (resulting byproducts carboxylic acid) with the BG fillers (Na^{2+}/Ca^{2+}) during degradation, leading to the formation of metal carboxylate salts (e.g., calcium/sodium lactate or acetate), which typically decompose in the temperature range of 300–400 °C [72-75]. The above findings demonstrate that the incorporation of BG fillers significantly reduces the earlier thermal stability of PLA/PCL/BG composites, indicating the catalytic degradation effect of BG fillers on the polymer matrix under high-temperature test conditions. The

catalytic degradation effect of BG fillers could well explain the change in the peak at 350 °C with increase of BG content. At lower BG content (5 wt%), during degradation, the BG fillers can be completely consumed by PLA/PCL polymer matrix, so no BG particles left can catalyze the PLA degradation. With increasing BG content to 10-15 wt%, the BG fillers cannot be completely consumed by PLA/PCL polymer matrix, and there are the BG particles left, which of course can cause the catalytic degradation of PLA (reducing PLA thermal stability).

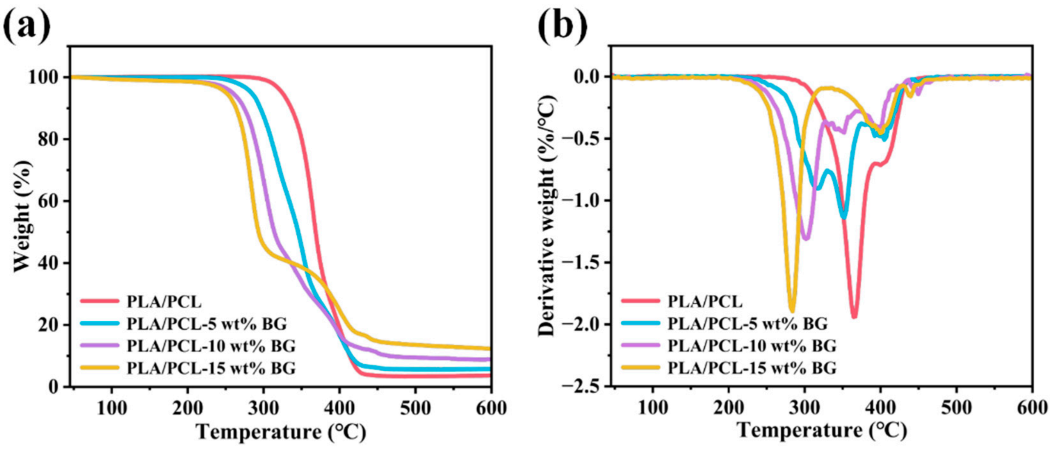

Figure S1 The TG (a) and DTG (b) curves of pure PLA/PCL blend and PLA/PCL/BG composites with various BG content (PCL content was fixed at 30 wt%).

References (Supporting references are numbered as [70], [71]... in main text Ref.[70-75])

[70] Liu, X.; Khor, S.; Petinakis, E.; Yu, L.; Simon, G.; Dean, K.; Bateman, S. Effects of hydrophilic fillers on the thermal degradation of poly(lactic acid). Thermochimica Acta. **2010**, 509, 147-151. https://doi.org/10.1016/j.tca.2010.06.015.

[71] Sivalingam, G.; Madras, G. Thermal degradation of binary physical mixtures and copolymers of poly(ε-caprolactone), poly(d, l-lactide), poly(glycolide). Polymer Degradation and Stability. **2004**, 84, 393-398. https://doi.org/10.1016/j.polymdegradstab.2003.12.008.

[72] Pielichowski, K; Njuguna, J.J.L.U. In Thermal Degradation of Polymeric Materials. Rapra Tech. **2005**, 80-100.

[73] Abdullah, N.; Kamarazaman, Z. Copper (II) Mixed Carboxylates As Metal-Containing Ionic Liquids. AIP Conf. **2009**, 1136, 361-365. https://doi.org/10.1063/1.3160164.

[74] Seesanong, S.; Wongchompoo, Y.; Boonchom, B.; Sronsri, C.; Laohavisuti, N.; Chaiseeda, K.; Boonmee, W. Economical and Environmentally Friendly Track of Biowaste Recycling of Scallop Shells to Calcium Lactate. ACS Omega. **2022**, 7, 14756-14764. https://doi.org/10.1021/acsomega.2c00112.

[75] Landoll, M.P.; Holtzapple, M.T. Kinetics study of thermal decomposition of sodium carboxylate salts. Biomass and Bioenergy. **2012**, 45, 195-202. https://doi.org/10.1016/j.biombioe.2012.06.005.
